# Human personality reflects spatio-temporal and time-frequency EEG structure

**DOI:** 10.1371/journal.pone.0197642

**Published:** 2018-09-07

**Authors:** Vladimir A. Maksimenko, Anastasia E. Runnova, Maksim O. Zhuravlev, Pavel Protasov, Roman Kulanin, Marina V. Khramova, Alexander N. Pisarchik, Alexander E. Hramov

**Affiliations:** 1 Research and Education Center “Artificial Intelligence Systems and Neurotechnologies”, Politehnicheskaya Str., 77, 410054 Saratov, Russia; 2 Saratov State University, Astrakhanskaya Str., 83, 410012 Saratov, Russia; 3 Center for Biomedical Technology, Technical University of Madrid, Campus Montegancedo, 28223 Pozuelo de Alarcón, Madrid, Spain; University of Wuerzburg, GERMANY

## Abstract

The reliable and objective assessment of intelligence and personality has been a topic of increasing interest of contemporary neuroscience and psychology. It is known that intelligence can be measured by estimating the mental speed or velocity of information processing. This is usually measured as a reaction time during elementary cognitive task processing, while personality is often assessed by means of questionnaires. On the other hand, human personality affects the way a subject accomplishes elementary cognitive tasks and, therefore, some personality features can define intelligence. It is expected that these features, as well as mental abilities in performing cognitive tasks are associated with the brain’s electrical neural activity. Although several studies reported correlation between event-related potentials, mental ability and intelligence, there is a lack of information about time-frequency and spatio-temporal structures of neural activity which characterize this relation. In the present work, we analyzed human electroencephalograms (EEG) recorded during the performance of elementary cognitive tasks using the Schulte test, which is a paper-pencil based instrument for assessing elementary cognitive ability or mental speed. According to particular features found of the EEG structure, we divided the subjects into three groups. For subjects in each group, we applied the Sixteen Personality Factor Questionnaire (16PF) to assess the their personality traits. We demonstrated that each group exhibited a different score on the personality scale, such as warmth, reasoning, emotional stability and dominance. Summing up, we found a link between EEG features, mental abilities and personality traits. The obtained results can be of great interest for testing human personality to create automatized intelligent programs which combine simple tests and EEG measurements for real estimation of human personality traits and mental abilities.

## Introduction

Finding a relation between human intelligence and personality is an important challenge of cognitive neuroscience and psychology. In psychology, these two terms are often studied separately; while intelligence is considered as a cognitive process, personality is defined as a non-cognitive one. However, the difference between these concepts is not so evident, because many personality traits have cognitive attributes and some of them are closely related to intelligence.

It is well known that intelligence can be estimated by measuring the mental speed or, in other words, the speed of information processing [1]. For this purpose, the reaction time to perform elementary cognitive tasks (ECTs) was studied [2, 3]. A popular type of the ECT is the so-called paper-and-pencil test [2]. The simplest ECTs are based on the *Hick paradigm* [4] which demonstrates the existence of a linear relationship between the amount of information to be processed by the subject and the reaction time. The latter can be estimated using *Sternberg memory scanning task* [5], according to which the reaction time increases linearly with the memory set size. A similar idea underlies the *letter matching paradigm* [6] which associates reaction time with the speed of lexical access.

There exists a direct correlation between mental speed and mental abilities (intelligence), i.e., more intelligent individuals exhibit lower reaction time and therefore higher speed of information processing. It was clearly demonstrated by Neubauer and Knorr [2], who measured the speed of information processing using Sternberg’s short term memory scanning and Posner’s letter matching, and compared them with the level of psychometric intelligence estimated via the *Berlin model of intelligence structure* [7].

Personality is usually assessed by means of questionnaires. Among them, the most popular are 16 Personality Factors questionnaire [8] and Big Five Questionnaire [9]. Similarly to intelligence, personality traits can also be estimated based on the speed of information processing. The correlation between personality traits and mental speed was first described in 1967 by H. J. Eysenck [10]. Later, in 1998 Soĉan and Bucik discovered a relationship between the speed of information processing and two major personality dimensions, extraversion and neuroticism [11]. In order to estimate the speed of information processing, they used the Hick reaction time paradigm [12] and the Sternberg’s short-term memory scanning paradigm [13]. Extraversion and its components were assessed by means of the Advanced Progressive Matrices, 16 Personality Factors questionnaire, and Big Five Questionnaire. The authors reported that different subdimensions of extraversion and neuroticism, which include dynamic, surgent and impulsive behavioural aspects, have different relationships with the speed of information processing measures. Particularly, in the case of extraversion, these subdimensions were found to be strongly correlated with mental speed, whereas in the case of neuroticism, they concerned ego strength and emotional control.

Although the relationship between personality, intelligence and mental speed has been reported many years ago, there is a concern whether or not ECTs are associated with complex factors of human personality [3]. In addition, in spite of low complexity of ECTs, they induce several complex cognitive processes in the brain, such as attention, perception, decision making, etc. [14]. Therefore, a study of the relationship between personality factors and mental speed requires consideration of the brain response to ECTs based on the detailed analysis of neurophysiological brain activity [3]. One of the first approaches to this problem was the diffusion model which decomposes information processing and decision making in performing the ECTs [15, 16]. According to Lerche et al. [17], the diffusion model is sensitive to the variation in the number of trials. This means that the robustness of the parameter estimation increases asymptotically with the number of trials to measure an experimental effect; crucially, however, there are many other trials needed to reliably measure individual differences [17–19].

Another very promising approach to firmly extract cognitive components associated with mental speed is the use of information about electrical brain activity (EEG). This approach was first implemented by Houlihan et al. [20], who recorded event-related potentials (ERP) during the Stemberg memory-scanning task. By analyzing ERP, they associated the latency of the P300 component with relative speed of information processing. As a result, they obtained negative association between ERP latencies and mental abilities. Later, ERP were used by Schubert et al. [3], who decomposed information-processing components in different ECTs. They found that association between ERP latencies and intelligence is mediated by reaction times. Recently, Euler et al. [21] considered ERP using the Hick paradigm in more detail. They analyzed four ERP components recorded with high spatial resolution with a 64-channel electrode cap, and found several ERP features strongly correlated with decision time, as well as the relation between IQ and P2 component amplitudes.

According to the above results, there exist particular EEG features which allow one to estimate not only the speed of information processing correlated with intelligence, but also other markers associated with personality traits. It should be noted that the latter were searched yet in 1973 by Edwards and Abbott [22], who analyzed EEG during resting states. However, their attempt was unsuccessful since personality could not be revealed when a person was at rest. Until now, this problem remains open. According to the recent review [23], the conclusions of the ERP studies of personality were contradictory, probably caused by differences in experimental protocols, sample size, and subject age. Other methods were only focused on the EEG power spectrum analysis. Having summarized, we have to note that the use of EEG features for assessment of personality traits and mental abilities still remains an exciting challenge of cognitive neuroscience. Although several studies reported the correlation between the ERP structure, mental abilities, and intelligence, there is a lack of information about time-frequency and spatio-temporal structures of neural activity underlying human intelligence and personality.

In the present work, we analyze relations between features of time-frequency and spatio-temporal structures of electrical brain activity, personality traits, and mental abilities. We record multichannel EEGs of subjects during performing the Schulte-table test. According to the revealed EEG features, we divide the subjects into three group. For subjects in each group, we estimate measures characterized subject’s mental ability during his/her accomplishing the Schulte test, namely, work efficiency (WE), work warming-up (WU), and psychological stability (PS). All these factors significantly differ in the groups. In order to measure personality traits, we use the Sixteen Personality Factor Questionnaire (16PF) [24, 25]. Having compared the results of personality description in the groups, we find that each group exhibits statistically different scores of personality scales, such as warmth, reasoning, emotional stability, and dominance. According to the obtained results, we conclude that there exists a relation between EEG features, mental abilities, and personality traits.

## Materials and methods

### Participants

Twenty two conditionally healthy men (33 ± 7 years), right-handed, amateur practitioners of physical exercises, and non-smokers participated at the experiment. All of them were asked to maintain a healthy life regime with an 8-hrs night rest during 48 hrs prior the experiment. All volunteers provided informed written consent before participating in the experiment. The experimental procedure was performed in accordance with the Helsinki’s Declaration and approved by the local Ethics Committee of the Yuri Gagarin State Technical University of Saratov.

### Experimental procedure

The experiments were carried out with each subject independently. The participants were previously informed about experimental conditions, but not about the experimental procedure, that was approved by the local Ethics Committee. The experimental research was conducted by independent researchers of various specializations and included two separate stages for each volunteer.

#### Personality traits assessment

For every participant, a personality multi-factor profile was described on the base of both the Sixteen Personality Factor Questionnaire (16PF) [24, 25] and a personal interview with an experienced psychologist. The 16PF contained 185 items organized into 16 primary factor scales and was adapted for Russian language and cultural context features [26–30]. We used the fully automated version of the 16PF, i.e., no paper-and-pencil materials were used. In this automated version, the items appeared on the screen one by one. There was the option to return to the immediately preceding item to correct inadvertent keying errors. However, the participant was not able to browse through the items. The program saved raw scale scores for every test and item responses.

#### Mental abilities assessment

We estimated cognitive abilities, such as cognitive tempo and preservation of attention during the process of elementary cognitive task accomplishing. In general, elementary cognitive abilities can be measured with the help of the d2 sustained-attention test or the Zahlen-Verbindungs-Test (ZVT) (in English “Number Connection Test”). d2 is a well-known neurophysiological tool for estimation of selective and sustained attention and visual scanning speed [19]. This is a paper-and-pencil test when the participant is asked to cross out any letter “d” with two marks above or below it in any order. The surrounding distractors are usually similar to the target stimulus, e.g., a “p” with two marks or a “d” with one or three marks. As distinct from the d2 test, ZVT is a digital-symbolic test, developed by Oswald and Roth in 1987 [31]. This test is based on the well-known principle of trail-making tests, but has a more solid theoretical basis. ZVT consists of four matrices of randomly arranged numbers (from 1 to 90) which the participant has to connect by drawing lines from number to number in an ascending order [2].

**Fig 1:**
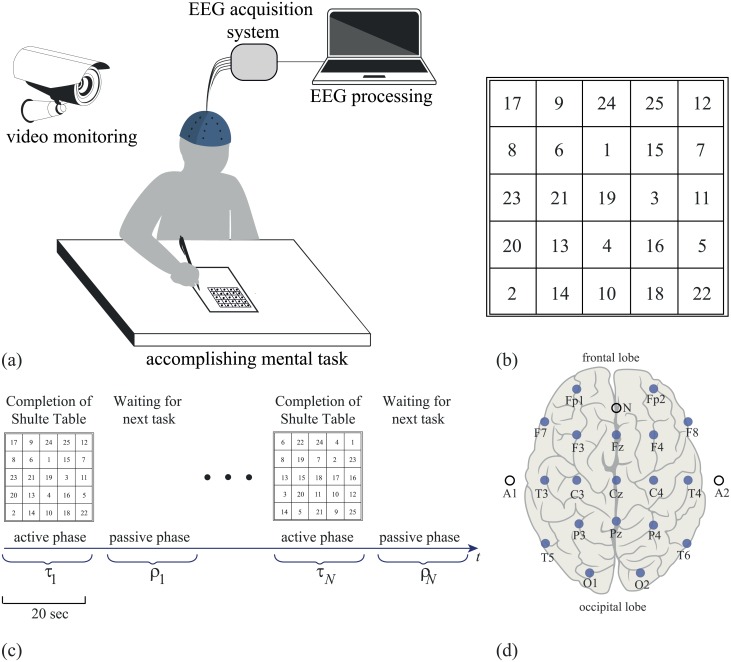
Experimental design. (a) Illustration of the experimental procedure. (b) Typical Schulte 5 × 5 table. (c) Experimental design: completion of *R* Schulte tables; *i*-th active phase of length *τ*_*i*_ followed by *i*-th passive phase of length *ρ*_*i*_ (waiting and preparing for the next task). (d) Layout of EEG electrodes arranged according to standard international 10–20 system.

In our study, we use the Schulte test, a simplified version of ZVT, widely used in Russia. Similarly to ZVT, the Schulte test consists of matrices of 5 × 5 randomly arranged numbers from 1 to 25 (see Fig 1(a) and 1(b)). Unlike ZVT, the subjects are asked to find numbers in a descending order. The participants have to find first the largest number (25), then the next largest number (24), etc., up to 1. During the task accomplishing, the subjects should not connect the respective cells by drawing a line, but only point each found number with a pencil.

Experiments were carried out during the first half of the day at a specially equipped laboratory where the volunteer was sitting comfortably. The influence of external stimuli, such as extraneous sounds and bright light, was minimized as much as possible. All participants had to complete *R* = 5 tables under direct supervision of a professional psychologist. The process of each table accomplishing was called an *active* experimental phase (Fig 1(c)). For every *i*-th active experimental phase, the completion time *T*_*i*_ was registered. Between the active phases, each volunteer had a short resting interval referred to as a *passive* experimental phase. The experimental design is shown in Fig 1(c).

It should be noted that by strictly adhering to standardized rest breaks, it is ensured that the same construct is measured throughout the experimental blocks, by preventing the performance decline originated from an increase in the probability of attentional lapses [32]. We consider the strict rest break schedule as a strength of our experimental design, because this aspect is often neglected in experimental personality research. However, according to Steinborn and Huestegge [32], the reaction time can be affected by the break, i.e., the reaction time of continuous mental arithmetic tasks increases if one does not adhere to the rest break regime.

It is known, that a stress state should be assessed in performance settings. For this purpose the Dundee Stress State Questionnaire (DSSQ) is usually used [33, 34]. In our study, we assessed the subject’s condition with the help of a subjective evaluation by a psychologist and at the same time by processing the Minnesota Multiphasic Personality Inventory (MMPI) questionnaire adapted for Russian-speaking subjects [35, 36]. Interviewing and testing the subjects showed their fairly calm states without pronounced stress components during the experiment.

During the experimental session, the EEG signals of the brain activity were recorded. The multi-channel EEG data were acquired by using the amplifier BE Plus LTM manufactured by the EB Neuro S.P.A., Italy (www.ebneuro.com). The data from 19 electrodes with two reference electrodes (A1 and A2) were recorded with a 8-kHz sampling rate using a standard monopolar method. Adhesive Ag/AgCl electrodes attached to a special pre-wired head cap were used. The ground electrode N was located above the forehead, while two reference electrodes A1 and A2 were located on the mastoids. The EEG signals were filtered by a band-pass filter with cut-off points at 1 Hz (HP) and 300 Hz (LP), and a 50-Hz Notch filter. During the experiment, a video synchronized with the EEG equipment was recorded. The video recording was processed manually and the time moments corresponding to starting and finishing points for each task were extracted. Then, the EEG recordings were divided into different epochs according to the obtained temporary protocol of the experiment. We extracted the EEG data corresponding to the active and passive experimental phases. The length of the active phases was varied from 30 to 50 seconds depending on the task completion speed, while the length of the passive phases was set to 10 seconds.

### The analysis of psycho-diagnostic tests

The current analysis of the 16PF answered items was based on sixteen personality scales: Warmth (reserved vs warm), Emotional Stability (reactive vs emotionally stable), Dominance (deferential vs dominant), Liveliness (serious vs lively), Rule-Consciousness (expedient vs rule-conscious), Social Boldness (shy vs socially bold), Sensitivity (utilitarian vs sensitive), Vigilance (trusting vs. vigilant), Abstractness (grounded vs abstracted), Privateness (forthright vs private), Apprehension (self-assured vs apprehensive), Openness to Change (traditional vs open to change), Self-Reliance (group-oriented vs self-reliant), Perfectionism (tolerates disorder vs perfectionistic), and Tension (relaxed vs tense). All these scales were estimated for every participant.

The Schulte tables are frequently used as a psychodiagnostic test for studying properties of human attention. This is one of the most objective methods to determine working effectiveness and ability, as well as resistance to external interference. The time *τ*_*i*_ of the *i*-th table completion was used to evaluate three standard test personal criteria: (1) work efficiency **WE** (the arithmetic mean of the table completion time), (2) warming-up work indicator **WU** (the ratio of the working time for the first table to **WE**), and (3) psychological stability **PS** (the ability to sustain the operational activity for a long time). These criteria are described by the following formulas:

$$\begin{array}{c} \text{WE}=\frac{\tau_{1}+\tau_{2}+\cdots +\tau_{R}}{R}, \end{array}$$ (1)

$$\begin{array}{c} \text{WU}=\frac{\tau_{1}}{\text{WE}}, \end{array}$$ (2)

$$\begin{array}{c} \text{PS}=\frac{\tau_{R-1}}{\text{WE}}. \end{array}$$ (3)

The work efficiency illustrates the attention consistency and performance. The resulted **WU** close to or lower than 1 indicates good warming-up, while 1 and higher means that the subject needs longer preparation time (warm-up) for the main work. The **PS** values close to 1.0 and smaler indicate a good psychological stability.

Since all tables are different, the learning effect is considered to be almost non-existent for the Schulte test. At the same time, the random arrangement of the numbers was chosen in such a way that the distance between the numbers in all presented tables exhibited a similar distribution, i.e., all tables have the same complexity. The experience shows that mentally healthy subjects spend from 30 to 50 seconds for one table, and usually the value of **WE** (the arithmetic mean of the table completion times given by Eq 1) is about 40–42 seconds. A decrease in the value of **WE** indicates good person’s ability to fix attention. Normally, the adult subject takes approximately equal time to accomplish every table. It is characterized by the degree of his/her workability (**WU**) and psychic stability (**PS**) (endurance) equal to 1.0. A strong deviation of **WU** and **PS** from 1.0 towards higher values indicates a decrease in workability and psychic stability, respectively. At the same time, a decrease in the **PS** characteristics with **WU** close to 1.0 can mean a high learning ability of the person who successfully improves his ability in performing the testing task.

Previously, the Schulte test was used for studying the correlation between the ERP structure and individual peculiarities of attention [37]. It was shown that the amplitudes of P2 and N1-P2 components of ERP, related to perception, negatively correlated with the **WE** index. Higher amplitudes indicate the subject’s ability to perform the cognitive task faster and therefore a higher level of voluntary attention. The amplitudes of P300 waves negatively correlate with **WE**, i.e., the stronger the attention, the higher the P300 magnitude.

### EEG analysis

We analyzed the EEG signals recorded by 19 electrodes placed on the standard positions of the 10–20 international system [38] (see Fig 1(d)), using the continuous wavelet transform. Before applying the wavelet analysis, we reduced large-amplitude artifacts in frontal cortex caused by eye blinks and movement. For this purpose, the EEG data from each electrode was processed via the Gram-Schmidt transformation using registered electrooculographic (EOG) signals (see [39] for details). Similar to [39], the EOG signals were recorded by two pairs of electrodes placed above (or below) the eyes and on the sides of the eyes.

The wavelet energy spectrum $E^{n}\left(f,t\right)=\sqrt{W_{n}{\left(f,t\right)}^{2}}$ was calculated for each EEG channel *X*_*n*_(*t*) in the frequency range *f* ∈ [1, 40] Hz. Here, *W*_*n*_(*f*, *t*) is the complex-valued wavelet coefficients calculated as [40]

$$\begin{array}{c} W_{n}\left(f,t\right)=\sqrt{f}\int_{t-4/f}^{t+4/f}X_{n}\left(t\right)\psi^{*}\left(f,t\right)dt, \end{array}$$ (4)

where *n* = 1, …, *N* is the EEG channel number (*N* = 19 being the total number of channels used for the analysis) and “*” defines the complex conjugation. The mother wavelet function *ψ*(*f*, *t*) is the Morlet wavelet often used for the analysis of neurophysiological data, defined as [40]

$$\begin{array}{c} \psi \left(f,t\right)=\sqrt{f}\pi^{1/4}e^{j\omega_{0}f\left(t-t_{0}\right)}e^{f{\left(t-t_{0}\right)}^{2}/2}, \end{array}$$ (5)

where *ω*_0_ = 2*π* is the central frequency of the mother Morlet wavelet.

Energy spectrum *E*^*n*^(*f*, *t*) was considered separately in the following frequency bands: delta (1–4 Hz), theta (4–8 Hz), alpha (8–13 Hz), beta–1 (13–23 Hz), beta–2 (24–34 Hz), and gamma (34–40 Hz) [41].

For these bands the values of wavelet energy $E_{\delta }^{n}\left(t\right)$, $E_{\theta }^{n}\left(t\right)$, $E_{\alpha }^{n}\left(t\right)$, $E_{\beta_{1}}^{n}\left(t\right)$, $E_{\beta_{2}}^{n}\left(t\right)$, and $E_{\gamma }^{n}\left(t\right)$ for each *n*-th EEG channel were calculated as

$$\begin{array}{c} E_{\delta ,\theta ,\alpha ,\beta_{1},\beta_{2},\gamma }^{n}\left(t\right)=\frac{1}{\Delta f}\int_{f\in \delta ,\theta ,\alpha ,\beta_{1},\beta_{2},\gamma }E^{n}\left(f,t\right)df. \end{array}$$ (6)

As a result, we considered the percentage of the spectral energy distributed in these bands, and calculated coefficients

$$\begin{array}{c} e_{\delta ,\theta ,\alpha ,\beta_{1},\beta_{2},\gamma }^{n}\left(t\right)=E_{\delta ,\theta ,\alpha ,\beta_{1},\beta_{2},\gamma }^{n}\left(t\right)/E_{0}^{n}\left(t\right)\;\left(\times 100\%\right), \end{array}$$ (7)

where *E*_0_(*t*) was defined as the whole energy and calculated as

$$\begin{array}{c} E_{0}^{n}\left(t\right)=\frac{1}{\Delta f}\int_{1\text{Hz}}^{40\text{Hz}}E^{n}\left(f,t\right)df. \end{array}$$ (8)

Finally, to describe the ratio between high frequency and low frequency brain activity for each channel, we introduced coefficient *ε*^*n*^ defined as

$$\begin{array}{c} \varepsilon^{n}=E_{\text{HF}}^{n}/E_{\text{LF}}^{n}, \end{array}$$ (9)

where

$$\begin{array}{c} E_{HF}^{n}\left(t\right)=\frac{1}{\Delta f}\int_{f>10\text{Hz}}E^{n}\left(f,t\right)df, \end{array}$$ (10)

$$\begin{array}{c} E_{LF}^{n}\left(t\right)=\frac{1}{\Delta f}\int_{f<10\text{Hz}}E^{n}\left(f,t\right)df. \end{array}$$ (11)

The ratio between spectral energies in high and low frequency bands is often used to characterize attention and its stability. For instance, Liutsyuk et al. [42] found that the subjects with good working ability displayed relatively high values of the ratio between spectral energies of *β*_1_ and *θ* rhythms. Moreover, this ratio was greater in the right hemisphere, that probably indicated stronger contribution of neuronal activity in this hemisphere to provide watchfulness and stability of attention. The subjects who performed the test with higher accuracy had higher ratios mostly in the central and parietal regions of both hemispheres.

The coefficients *ε*^*n*^ were calculated for each EEG channel for both the active and the passive phases. The obtained values of *ε*_*n*_ were averaged over the channels located on the left and right hemispheres, defined respectively as

$$\begin{array}{c} \varepsilon_{\text{LH}}=\frac{1}{N_{\text{LH}}}\sum_{n}\frac{E_{HF}^{n}}{E_{LF}^{n}},\;n=\left\{\text{Fp}1,F3,F7,C3,T3,P3,T5,O1\right\},\;N_{\text{LH}}=8, \end{array}$$ (12)

$$\begin{array}{c} \varepsilon_{\text{RH}}=\frac{1}{N_{\text{RH}}}\sum_{n}\frac{E_{HF}^{n}}{E_{LF}^{n}},\;n=\left\{\text{Fp}2,F4,F8,C4,T4,P4,T6,O2\right\},\;N_{\text{RH}}=8. \end{array}$$ (13)

The obtained coefficients *ε*_LH_ and *ε*_RH_ quantify the electrical activity in the left and right hemispheres, respectively, at the sculp level. The degree of interhemispheric asymmetry of electrical activity is usually considered as a marker of physiologically adequate development, and often associated with enhanced cognition [43]. There is also an opinion that an abnormal lateralization is associated with psychiatric disorders, such as autism [44] and depression [45]. The analysis of interhemispheric differences in electrical brain activity is often used to study auditory and visual attention [46, 47]. In particular, the interhemispheric differences in the spectral power of EEG rhythms were recently used by Luschekina et al. [48] for mental ability assessment in children with autism spectrum disorders. In the recent study of Sartarnecchi et al. [49], intelligence-related differences in the asymmetry of brain activity were reported.

### Clustering analysis

**Fig 2:**
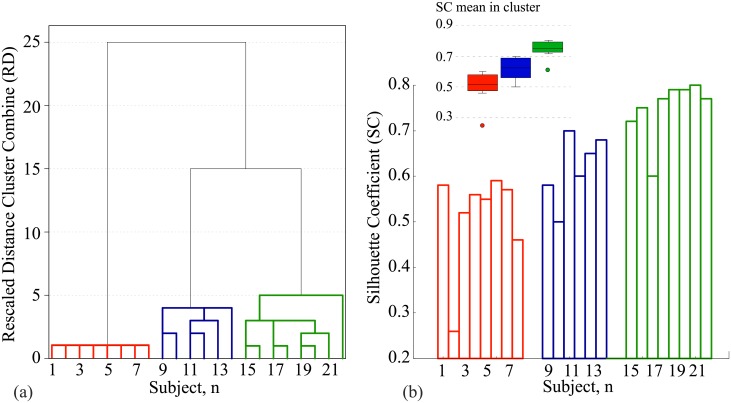
Clustering analysis. (a) Dendrogram illustrating the results of hierarchical clustering. (b) Silhouette coefficients calculated for all 22 subjects (histogram) and averaged over the subjects belonging to each of three clusters (boxes). Different clusters are marked with different colors.

In order to group the subjects according to the features of their electrical brain activity, we applied the cluster analysis based on the hierarchical clustering method [50]. This method allowed us to group the data into a tree of clusters [51]. Such hierarchical clustering is widely used by many researchers [50, 52] because it allows higher quality clustering than other methods, e.g., k-means. To characterize subject’s electrical brain activity, we performed the hierarchical cluster analysis using variables *ε*_*LH*_ and *ε*_*RH*_ calculated by Eqs (12) and (13) for both the active and the passive phases. We used SPSS statistics to perform the clustering. We chose the Squared Euclidean distance (default) method to determine the distance between the clusters and the Furthest Neighbour clustering method. The dendrogram in Fig 2 displays the results of clustering.

In Fig 2(a), we plot the rescaled distance (RD) (in the parameter space) between pairs or groups of subjects clustered in a particular step, estimated using a 0–25 scale. The hierarchic dendrogram allows one to trace backward or forward any individual subject or cluster of subjects at any level. The bigger the distance before two clusters joined, the larger the difference between these clusters. According to this, all participants were grouped into three clusters marked with different colors. After the clusters were formed, by analogy with [50] we evaluated their quality by computing the Silhouette Coefficient (SC) of the cluster [53]. SC is defined as a measure of how the objects in the same cluster are similar and different from the objects in other clusters. SC values ranges from -1 to +1, where +1 indicates that an object well matches other objects in its own cluster and poorly matches objects in neighboring clusters. If most objects in the cluster have high SC, then the clustering is appropriate, otherwise, the clustering is inappropriate [50]. SCs were calculated using Orange Software [54]. The histogram in Fig 2(b) displays the values of SC calculated for all subjects, while the inset box shows SC averaged over the subjects belonging to each of three clusters. One can see, that for all clusters the mean SC values exceed 0.51 and, therefore, the clustering can be judged as reasonable [55].

## Results and discussion

### EEG features

In order to analyze the features of electrical brain activity, we calculated the values of $e_{\delta ,\theta ,\alpha ,\beta_{1},\beta_{2},\gamma }^{n}\left(t\right)$ using Eq (7) for *n* = 1, …19 EEG channels. The obtained coefficients determine the percentage of the spectral energy belonging, respectively, to delta, theta, alpha, beta–1, beta–2, and gamma frequency bands, and characterize the degree of participation of the neural ensemble located in the vicinity of the *n*-th recording electrode, in generation of the corresponding type of activity [56].

**Fig 3:**
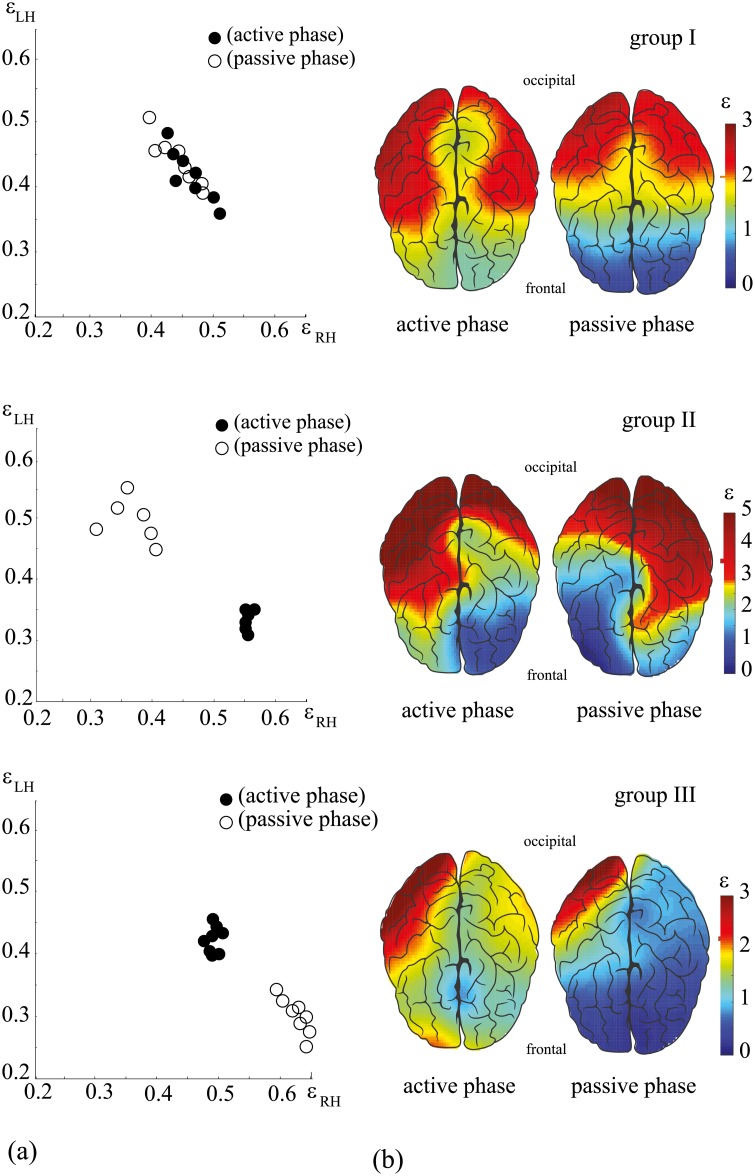
Three scenarios of cognitive activity during mental task processing. (a) Relation between energies of high- and low-frequency spectral components in the left (*ε*_LH_) and right (*ε*_RH_) hemispheres, calculated for active (closed dots) and passive (open dots) experimental phases. The distributions are shown for three subjects belonging to different groups. (b) Coefficient *ε* showing the relation between energies of high-and low-frequency spectral components, calculated for each EEG channel during active (left-hand columns) and passive (right-hand columns) phases. Groups I and III contain *n* = 8 subjects and group II contains *n* = 6 subjects.

Next, in order to describe neural dynamics in the left and right hemispheres, we considered coefficients *ε*_LH_ (12) and *ε*_RH_ (13) obtained by averaging coefficients *ε* calculated for EEG channels belonging to the left and right hemispheres, respectively. According to hierarchical clustering method (see Material and methods), the subjects can be promptly divided into three groups. In Fig 3(a), we plot the values *ε*_RH_ and *ε*_LH_ for each of the 22 participants in the active (closed dots) and passive (open dots) phases (each group is shown on separate subplot).

The source data containing the values of *ε*_RH_ and *ε*_LH_ for each subject are shown in Table 1. It is clearly seen from Fig 3(a) that the behavior of *ε*_RH_ and *ε*_LH_ in each group is different. In group I, *ε*_RH_ and *ε*_LH_ have practically the same values during the active and passive phases. In group II, the active phase is associated with an increase in high-frequency activity in the right hemisphere and the passive phase with an increase in high-frequency activity in the left hemisphere. In group III, the transition from the active to the passive phase is associated with a pronounced increase in *ε*_RH_ and a decrease in *ε*_LH_.

**Table 1:** EEG features revealed during active phase (accomplishing Schulte table) and passive phase, in terms of coefficients *ε*_LH,RH_ and *k*. *ε*_LH,RH_ is the ratio between high- and low-frequency activity in the left and right hemispheres and *k* = *ε*_RH_/*ε*_LH_ is the degree of hemispheric asymmetry.

Group	Subject	Active phase		Passive phase		Active phase	Passive phase
		*ε*_*RH*_	*ε*_*LH*_	*ε*_*RH*_	*ε*_*LH*_	*k*	*k*
I	1	0.471	0.422	0.460	0.417	1.11	1.10
	2	0.426	0.482	0.397	0.506	0.88	0.78
	3	0.510	0.358	0.484	0.390	1.42	1.23
	4	0.450	0.440	0.407	0.456	1.02	0.89
	5	0.440	0.451	0.425	0.466	0.97	0.92
	6	0.440	0.410	0.445	0.450	1.07	0.98
	7	0.470	0.404	0.450	0.432	1.17	1.04
	8	0.500	0.389	0.483	0.405	1.31	1.18
II	9	0.551	0.349	0.398	0.473	1.57	0.84
	10	0.551	0.323	0.406	0.448	1.70	0.90
	11	0.555	0.342	0.343	0.515	1.62	0.66
	12	0.565	0.350	0.314	0.470	1.61	0.65
	13	0.550	0.334	0.368	0.555	1.66	0.65
	14	0.560	0.319	0.386	0.513	1.80	0.74
III	15	0.497	0.443	0.604	0.324	1.12	1.86
	16	0.487	0.396	0.642	0.250	1.22	2.56
	17	0.490	0.451	0.590	0.340	1.08	1.73
	18	0.500	0.400	0.657	0.277	1.25	2.40
	19	0.485	0.400	0.635	0.294	1.21	2.17
	20	0.510	0.436	0.641	0.290	1.18	2.16
	21	0.491	0.430	0.623	0.317	1.13	2.00
	22	0.480	0.415	0.612	0.315	1.15	1.96

Fig 3(b) represents spatio-temporal brain activity in the units of *ε* in the active and passive phases for each of the three groups. In group I, the brain activity during the active phase is characterized by hemispheric symmetry, whereas in the passive phase, the hemispheric symmetry persists, although the spatio-temporal structure changes.

In group II, the spatio-temporal structure is significantly different. One can notice hemispheric asymmetry during both the active and the passive phases. However, the character of the asymmetry is different in these phases, namely, high-frequency activity prevails in the right hemisphere during the active phase and moves to the left hemisphere during the passive phase.

In group III, the subjects also exhibit hemispheric asymmetry during both the active and the passive phases. Unlike group II, the character of the asymmetry remains the same in both phases. As seen from Fig 3(b), the asymmetry in both phases manifests itself as a dominance of high-frequency activity in the right hemisphere. At the same time, the difference between the active and passive states reveals a decrease in *ε* in the right hemisphere during the transition from the active to passive phase.

In order to check whether or not the groups significantly differed from each other, we applied the multivariate analysis of variance (MANOVA). As a criterion for belonging to one of three groups, we chose a between-subjects factor (independent variable). On the other hand, the values of *ε*_RH_ and *ε*_LH_ calculated for the active and passive phases were considered as within-subjects factors (dependent variables). As a result of this analysis, we found significant differences between the groups. The multiple comparisons revealed significant differences across all factors, with an exception of *ε*_LH_ (*p* = 0.858) calculated in the active phase in groups 1 and 3.

**Fig 4:**
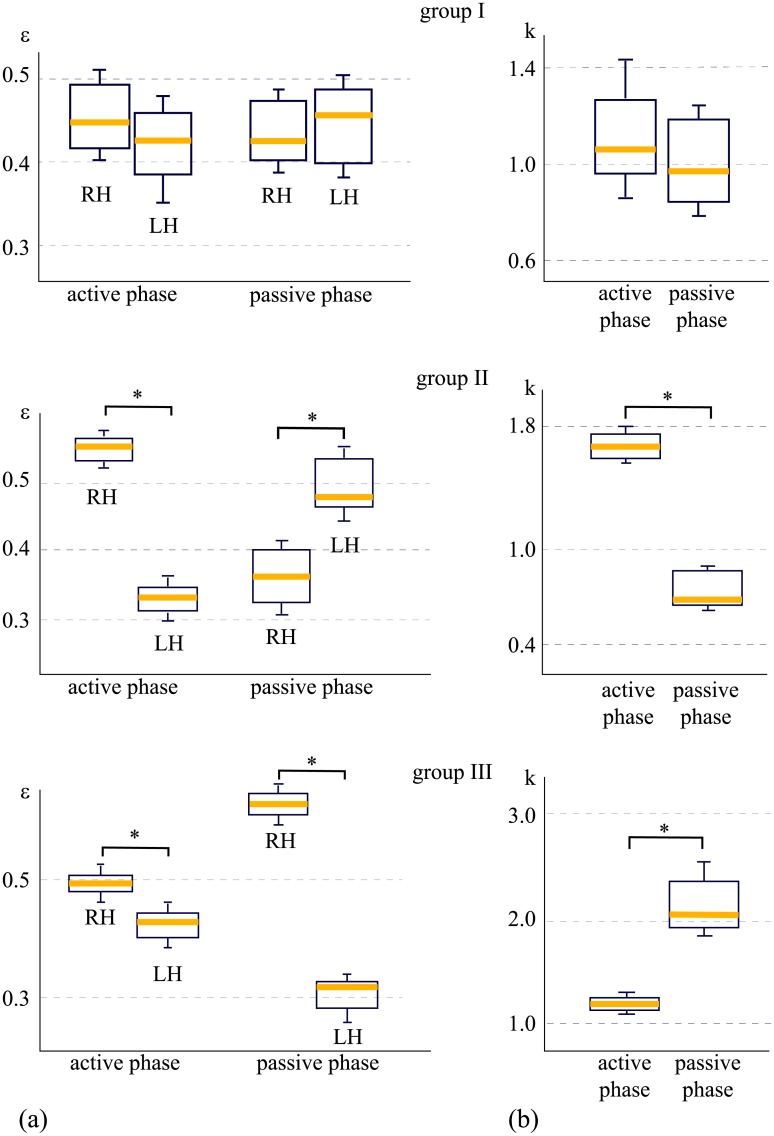
Statistical measures for three scenarios of cognitive activity. (a) Ratio *ε* between energies of high- and low-frequency spectral components calculated for EEG channels belonging to left (LH) and right (RH) hemispheres during active and passive phases. (b) Ratio *k* between values of *ε* calculated for left and right hemispheres during active and passive phases. Yellow bars, boxes, and whiskers indicate, respectively, medians, 25–75 percentiles, and outlines. Groups I and III contain *n* = 8 subjects and group II contains *n* = 6 subjects. *p < 0.05 via nonparametric Wilcoxon signed-rank test.

The distinguished features of brain activity during the active and passive phases, observed in three groups are shown in Fig 4(a). The horizontal yellow bars indicate the median of *ε* calculated for the left (LH) and right (RH) hemispheres during the active and passive phases. In group I, the values of *ε* remain practically the same for different hemispheres in both the active and passive phases (*p* = 0.123 and *p* = 0.889 via the nonparametric Wilcoxon signed-rank test (NPWSRT), *n* = 8). In group II, the active phase is characterized by a sharp increase in *ε* in the right hemisphere (median *ε*_RH_ > 0.5 vs median *ε*_LH_ < 0.35) (*p* < 0.05 via NPWSRT, *n* = 6). In the passive phase, the dynamics is reversed, namely, an increase in *ε* is observed in the left hemisphere (median *ε*_RH_ < 0.4 vs median *ε*_LH_ > 0.45) (*p* < 0.05 via NPWSRT, *n* = 6). Finally, in group III, during the active phase, *ε* in the right hemisphere is slightly higher than that in the left hemisphere (median *ε*_RH_ > 0.45 vs median *ε*_LH_ < 0.45) (*p* < 0.05 via NPWSRT, *n* = 8). During the passive phase, such a difference becomes greater (median *ε*_RH_ > 0.6 vs median *ε*_LH_ < 0.35) (*p* < 0.05 via NPWSRT, *n* = 8).

It is known that the completion of mental tasks is associated with changes in neural activity, which can be detected in the EEG power spectrum. The role of low-frequency **delta activity** in mental tasks was studied in [57], where the authors reported on increasing delta EEG activity during mental tasks, associated with enhancing attention. Later [58], a relation between delta-oscillations and the performance of mental tasks was also identified. On the other hand, earlier works [59, 60] highlighted an increase in **theta activity** during mental efforts. Recently, a change in the activity level in the low-frequency *θ*-band was used to evaluate the dynamics of mental workload [61].

The relation between **alpha activity** and the completion of mental tasks was demonstrated yet in 1984 by Osaka [62], who detected changes in the amplitude and location of the peak alpha frequency in the power spectrum. Later, a significant role of alpha activity in memory and cognitive processes was identified [63]. Changes in the energy of high-frequency brain rhythms are usually related to cognitive activity, in particular, mental task completion [64]. For instance, the account of **gamma activity** for classification of mental tasks improves the accuracy [65].

According to Fig 4(a), one can see that electrical brain activity in each group follows a particular scenario defined, on one hand, by the lateralization of the brain function, and on the other hand, by specific transitions between active and passive phases. In order to quantitatively describe the observed scenarios, we calculated *k* = *ε*_RH_/*ε*_LH_, which reflects a degree of hemispheric asymmetry. These values are plotted for each group in Fig 4(b). One can see that group I is characterized by hemispheric symmetry in active and passive phases, which remains unchanged during active-passive phase transition (Δ*k* ≈ 0), where Δ*k* = *k*_passive_ − *k*_active_. For other groups, asymmetry and transition are observed between active and passive phases, and plotted in terms of *k* which can be described as Δ*k* < 0 and Δ*k* > 0, respectively.

### Correlation between EEG features and mental abilities

The participants belonging to each of the three groups were subjected to psycho-diagnostic tests (see Methods). As a result, the values of **WE**, **WU**, and **PS**, which define the average time of task completion, average performance, and attention preservation, respectively, were estimated for each subject (see Table 2).

**Table 2:** Schulte performance in terms of work efficiency (WE), psychological stability (PS), and warming-up work indicator (WU).

Group	Subject	Work efficiency, WE, [second]	Psychological stability, PS	Warming-up work indicator, WU
I	1	33	0.90	0.85
	2	31	1.12	0.9
	3	35.8	0.92	0.87
	4	32.4	1.08	0.84
	5	34.25	1.17	0.89
	6	31.8	1.30	0.83
	7	35.21	0.99	0.89
	8	35.6	1.07	0.88
II	9	38.5	1	1.02
	10	39.9	0.91	1.01
	11	40.5	1.08	0.99
	12	41.2	1.1	0.92
	13	41	1.3	0.91
	14	40.3	1.04	0.92
III	15	35.2	1.15	0.92
	16	34	1.1	0.97
	17	31.3	1.29	0.84
	18	33.4	1.24	0.89
	19	34.2	1.3	0.92
	20	35.1	1.23	0.91
	21	30.2	1.32	0.93
	22	33	1.3	0.89

**Fig 5:**
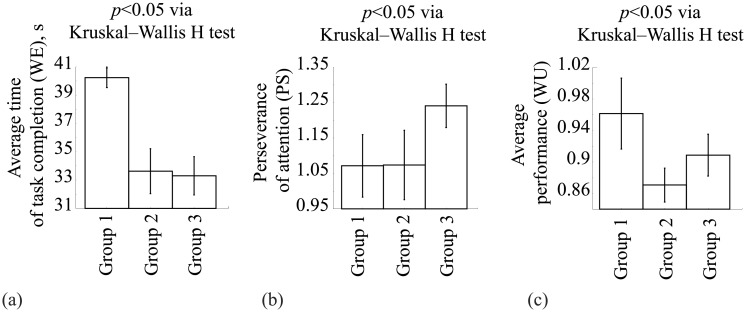
Results of psycho-diagnostic tests. Measures characterizing subject’s mental ability during accomplishing the Schulte test. (a) Average time of task completion **WE** (calculated via Eq 1, measured in seconds). (b) Perseverance of attention **PS** (calculated via Eq 3, measured in dimensionless units). (c) Average performance **WU** (calculated via Eq 2, measured in dimensionless units). The data are presented as mean±SD (SD being standard deviation).

The results of psycho-diagnostic tests are presented in Fig 5, where each subplot illustrates the values of **WE** (a), **PS** (b), and **WU** (c) for each of three groups. Data are shown as mean±SD. The differences in the results of the psycho-diagnostic tests were compared statistically between the groups of subjects. We applied the nonparametric Kruskal–Wallis H test for multiple independent samples for quantification of the change in the values of **WE**, **WU**, and **PS** across the groups. As a result, we obtained *p* < 0.05 for average performance **WU**, average time of task completion **WE**, and perseverance of attention **PS**.

The subjects from group I demonstrated bilateral EEG activity in both hemispheres during the Schulte tables tests. Simultaneously, these subjects demonstrated a medium-low efficiency when performing the task. For them, the average time of task completion was **WE** = 40.2 ± 0.68 seconds and the average performance was **WU** = 1.07 ± 0.08 (target value was 1). The perseverance of attention was high **PS** = 0.97 ± 0.045 (target value was 1). The subjects from this group could immediately perform unknown tasks and maintain their working efficiency at a relatively high rate, above a medium-low level. The psychological decryption of the tests included the remarks about the creativity in the test performance and fast switches to new tasks. In the personal test, such subjects had a pronounced tendency to work alone, high intellect, analytical mind, critical thinking, intolerance to uncertainty, and a delay in decision making. Moreover, they exhibited self-control, a lack of anxiety, a pronounced leadership, and a desire to dominate in the group. We hypothesize that the creativity and the attempt to optimize their work led to a decrease of their working efficiency.

The subjects from group II tried to develop a strategy to simplify the task performance. During the accomplishment of the first task, a maximum lateralization of high-frequency activity was present, i.e., the activity in the right hemisphere was much more pronounced. This means that during the first task, the strategy was not yet developed. During the next tasks, the burden in the right hemisphere in these subjects was reduced. As a result, the subjects from group II demonstrated higher working efficiency than the subjects from group I. The average time of task completion was **WE** = 33.6 ± 1.58 seconds. Persistence of attention **PS** = 0.86 ± 0.02 (target value was 1). The average performance was **WU** = 1.07 ± 0.09 (target value was 1). These subjects needed little time for adaptation and did not tire, being capable to effectively maintain a high working efficiency for a long time. Their personal profiles harmoniously combined high scores in intellect, emotional maturity, and self-control.

Unlike group II, the subjects from group III accomplished the task without any attempts to develop a strategy to simplify it. This was confirmed by the psychological test. Their working efficiency remained high; the average time of the task completion was **WE** = 33 ± 1.35 seconds. The perseverance of attention was **PS** = 0.9 ± 0.02 (target value was 1). The average performance was **WU** = 1.24 ± 0.06 (target value was 1). We assume that the subjects from this group have difficulties to maintain high working efficiency for prolong time. Their personal tests showed a pronounced preference to work alone with low self-control, intolerance to uncertainty, and a delay in decision-making, that can be manifested by anxiety. They also demonstrated high intellect, analytical mind, critical thinking, and a spirit for experimentation.

### Correlation with personality traits

**Fig 6:**
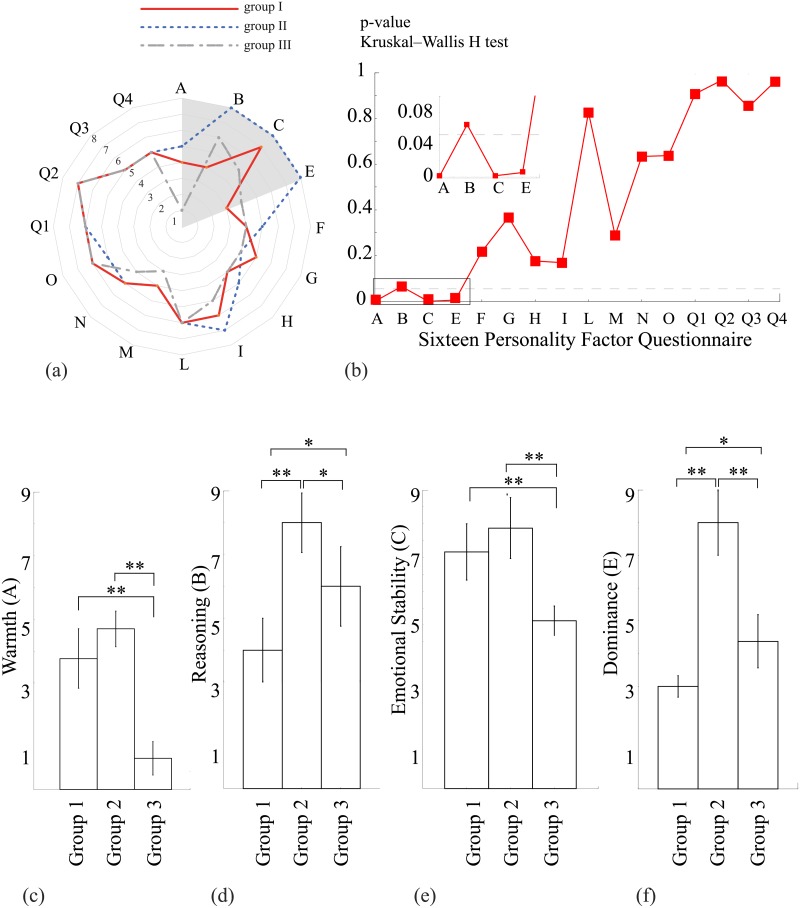
Sixteen Personality Factor Questionnaire. (a) Primary factors of 16PF Questionnaire, averaged over subjects in each group (group I—dotted line, group II—solid line, group III—dash-dotted line). The dashed area highlights the factors for which significant changes between the groups are observed. (b) *p*-values calculated for these groups for different factors of 16PF Questionnaire via Kruskal–Wallis H test for several independent samples. The insert shows in detail low *p*-values calculated for A, B, C, E factors. (c-e) Values of A, B, C, E calculated for three groups (data are shown as mean±SD). Groups I and III contain *n* = 8 subjects and group II *n* = 6 subjects, *p > 0.05, **p > 0.01 via nonparamentric Mann–Whitney U test.

The participants belonging to each of the three groups were subjected to the Cattell’s 16 Personality Factors Test. The diagram in Fig 6(a) shows the results of the Cattell’s 16 Personality Factors Test for three groups. The data are displayed as the values of all primary factors of the 16PF Questionnaire, averaged over all subjects in each group. One can see that most of the factors have similar values in each group. At the same time, for some factors the corresponding values vary significantly from one group to another. Among these factors, one can distinguish Warmth (A), Reasoning (B), Emotional Stability (C), and Dominance (E). In order to quantify the differences between groups in each of analyzed personality factors, we have applied nonparametric Kruskal-Wallis H test for multiple independent samples. The *p*-values, calculated for each of 16 personality scales are shown in Fig 6(b). One can see that for 4 factors (A, B, C, E) *p*-value is relatively small (*p* ≤ 0.05), while for other factors *p*-value is significantly large. According to this, we have considered these 4 factors in more detail and compare how they are differed within groups. We have applied nonparametric Mann–Whitney U test in order to statistically analyze the difference between factors in each pair of groups. As the result, we have found that group 1 and group 2 do not demonstrate a significant change in A (*p* = 0.218) and C (*p* = 0.39) factors. At the same time, these group are significantly different in B and E factors (*p* < 0.01). On the other hand, the differences between groups 1–3 and 2–3 are significant for all considered factors (Fig 6(c)–6(f)).

The diagram in Fig 6(a) shows the results of the Cattell’s 16 Personality Factors Test for three groups. The data are displayed as the values of all primary factors of the 16PF Questionnaire, averaged over all subjects in each group. One can see that most of the factors have similar values in each group. At the same time, for some factors the corresponding values vary significantly from one group to another. Among these factors, one can distinguish Warmth (A), Reasoning (B), Emotional Stability (C), and Dominance (E), which are tabled and compared with the results of the EEG study and the psycho-diagnostic test in Fig 6(b).

According to the results of personality classification based on the psycho-diagnostic test, different features of the EEG structure, namely, lateralization and the ratio between energy of high- and low-frequency waves, reflect different personal qualities. It is important to note, that while EEG activity varied among different groups, it represented the same scenario inside each group. A similar behavior was observed in psychological classification, where three groups of subjects with similar personal profiles were identified.

Usually, the majority of scientific publications which aimed to reveal the EEG signatures of the cognitive activity describe the scenario, which is repeated from one subject to another. At the same time, we show that the differences occurred from one subject to another, can also be systematized. Different scenarios of cognitive activity can be identified among the subjects depending on the personality.

Our results confirm the hypothesis raised by Vingiano and William [66] about the existence of a relation between brain hemisphericity and personality. Our results are also in accordance with the work of [67], where the anxiety-related properties of personality, estimated via Cattel’s technique, were shown to correlate with the spectral power density (SPD) of EEG rhythms, in particular, beta–1 and beta–2. The authors claimed that the intense beta EEG rhythm correlates with highly situational and individual anxieties. At the same time, an individual’s emotional stability was found to be related to the alpha rhythm power.

Thus, the obtained results provide new knowledge in understanding the features of human personality by analyzing the relation between spatio-temporal and time-frequency EEG structure.

It should be noted, that in general, in order to firmly make predictions about personality, much larger sample size is required. At the same time, in our study we tried to create the most homogeneous group of volunteers in order to eliminate the inevitable impact of additional, poorly considered factors, on the results of our assessments. Further expansion of the group of subjects to randomly selected persons (with different physical condition, gender, educational level, etc.) is planned. This should be accomplished, first, by an increase in the number of subjects, and, second, by an addition of various psychological testing techniques and personal psychological interviews of each subject, made by a psychologist.

## Conclusion

We have analyzed the correlation between neurophysiological processes and personal characteristics during complicated mental tasks using a series of simple psycho-diagnostic tests to study human personality. To solve these task, we considered spatio-temporal and time-frequency structures of multichannel EEGs in humans, who completed the Schulte tables. We have found that EEG activity during the mental tasks varied from one subject to another. Based on the EEG data analysis, we divided all subjects into three group depending on particular features of their electrical brain activity. At the same time, all subjects performed psycho-diagnostic tests in order to estimate their mental abilities, e.g., work efficiency, warming-up work indicator, and psychological stability during the task accomplishing. As a result, we have found that the scores which defined mental abilities were significantly different in the groups. Finally, we applied Sixteen Personality Factor Questionnaire (16PF) to assess subjects’ personality traits and found that different groups exhibited different scores for such personality scales as warmth, reasoning, emotional stability, and dominance. Summing up, we demonstrated the relation between EEG features, mental abilities, and personality traits.

We believe that our results can help in testing and diagnostic of personal skills and abilities to perform complex operational tasks. On the base of our findings, automatic intelligent systems can be developed to examine subject’s strong and weak points for high demanding purposes.

## References

[1] Sheppard LD, Vernon PA. Intelligence and speed of information-processing: A review of 50 years of research. Personality and Individual Differences. 2008;44(3):535–551. 10.1016/j.paid.2007.09.015

[2] Neubauer AC, Knorr E. Three paper-and-pencil tests for speed of information processing: Psychometric properties and correlations with intelligence. Intelligence. 1998;26(2):123–151. 10.1016/S0160-2896(99)80058-0

[3] Schubert AL, Hagemann D, Voss A, Schankin A, Bergmann K. Decomposing the relationship between mental speed and mental abilities. Intelligence. 2015;51:28–46. 10.1016/j.intell.2015.05.002

[4] Hick WE. On the rate of gain of information. Quarterly Journal of Experimental Psychology. 1952;4(1):11–26. 10.1080/17470215208416600

[5] Sternberg S. Memory-scanning: Mental processes revealed by reaction-time experiments. American Scientist. 1969;57(4):421–457. 5360276

[6] Posner MI, Mitchell RF. Chronometric analysis of classification. Psychological Review. 1967;74(5):392–409. 10.1037/h0024913 6076470

[7] Jäger AO, Süß HM, Beauducel A. Berliner Intelligenzstruktur-Test: BIS-Test. Hogrefe; 1997.

[8] Cattell RB, P Cattell HE. Personality structure and the new fifth edition of the 16PF. Educational and Psychological Measurement. 1995;55(6):926–937. 10.1177/0013164495055006002

[9] Digman JM. Personality structure: Emergence of the five-factor model. Annual Review of Psychology. 1990;41(1):417–440. 10.1146/annurev.ps.41.020190.002221

[10] Eysenck H. The biological basis of personality. Routledge; 2017. 10.1038/1991031a0 14066934

[11] SoCan G, Bucik V. Relationship between speed of information-processing and two major personality dimensions-extraversion and neuroticism. Personality and Individual Differences. 1998;25:35–48. 10.1016/S0191-8869(98)00031-2

[12] Neubauer AC, Bucik V. The mental speed-IQ relationship: unitary or modular? Intelligence. 1996;22(1):23–48. 10.1016/S0160-2896(96)90019-7

[13] Sternberg RJ, Ruzgis P. Personality and Intelligence. Cambridge University Press; 1994.

[14] Donders FC. On the speed of mental processes. Acta Psychologica. 1969;30:412–431. 10.1016/0001-6918(69)90065-1 5811531

[15] Ratcliff R. A theory of memory retrieval. Psychological Review. 1978;85(2):59 10.1037/0033-295X.85.2.59

[16] Lerche V, Voss A. Retest reliability of the parameters of the Ratcliff diffusion model. Psychological Research. 2017;81(3):629–652. 10.1007/s00426-016-0770-5 27107855

[17] Lerche V, Voss A, Nagler M. How many trials are required for parameter estimation in diffusion modeling? A comparison of different optimization criteria. Behavior Research Methods. 2017;49(2):513–537. 10.3758/s13428-016-0740-2 27287445

[18] Miller J, Ulrich R. Mental chronometry and individual differences: Modeling reliabilities and correlations of reaction time means and effect sizes. Psychonomic bulletin & review. 2013;20(5):819–858. 10.3758/s13423-013-0404-5 23955122

[19] Steinborn MB, Langner R, Flehmig HC, Huestegge L. Methodology of performance scoring in the d2 sustained-attention test: cumulative-reliability functions and practical guidelines. Psychol Assess. 2018;30(3):339–357 10.1037/pas0000482 28406669

[20] Houlihan M, Stelmack R, Campbell K. Intelligence and the effects of perceptual processing demands, task difficulty and processing speed on P300, reaction time and movement time. Intelligence. 1998;26(1):9–25. 10.1016/S0160-2896(99)80049-X

[21] Euler MJ, McKinney TL, Schryver HM, Okabe H. ERP correlates of the decision time-IQ relationship: The role of complexity in task-and brain-IQ effects. Intelligence. 2017;65:1–10. 10.1016/j.intell.2017.08.003

[22] Edwards AL, Abbott RD. Measurement of personality traits: Theory and technique. Annual review of psychology. 1973;24(1):241–278. 10.1146/annurev.ps.24.020173.001325 4146758

[23] Roslan NS, Izhar LI, Faye I, Saad MNM, Sivapalan S, Rahman MA. Review of EEG and ERP studies of extraversion personality for baseline and cognitive tasks. Personality and Individual Differences. 2017;119:323–332. 10.1016/j.paid.2017.07.040

[24] Cattell RB. The scree test for the number of factors. Multivariate Behavioral Research. 1966;1(2):245–276. 10.1207/s15327906mbr0102_10 26828106

[25] Conn SR, Rieke ML. 16PF fifth edition technical manual. Institute for Personality and Ability Testing, Incorporated; 1994.

[26] Shmelev AG, Pokhilko VI, Soloveichik AS, Burmistrov IV. Questionnaire of 16 personality factors R. Cattell In: Workshop on psychodiagnostics. Specific psychodiagnostic techniques. Moscow: Published by Moscow University; 1989.

[27] Grigorenko EL. Multicultural Psychoeducational Assessment. Springer Publishing Co Inc, New York, United States; 2009.

[28] Kuznetsova J, Babyonyshev M, Reich J, Hart L, Grigorenko E. The acquisition of universal quantifiers in Russian. Proceedings of the 2nd Conference on Generative Approaches to Language Acquisition—North America. 2007; p. 224–232.

[29] Grigorenko EL, LaBuda MC, Carter AS. Similarity in general cognitive ability, creativity, and cognitive styles in a sample of adolescent Russian twins. Acta Geneticae Medicae et Gemellologiae. 1992;(41):65–72. 10.1017/S000156600000252X 1488859

[30] Sternberg RJ, Grigorenko EL. Ability Testing Across Cultures; 2008.

[31] Oswald WD, Roth E. Der Zahlen-Verbindungs-Test: (ZVT); ein sprachfreier Intelligenz-Test zur Messung der “kognitiven Leistungsgeschwindigkeit”; Handanweisung. Verlag für Psychologie Hogrefe; 1987.

[32] Steinborn MB, Huestegge L. A walk down the lane gives wings to your brain. Restorative benefits of rest breaks on cognition and self-control. Applied Cognitive Psychology. 2016;30(5):795–805. 10.1002/acp.3255

[33] Matthews G, Campbell SE, Falconer S, Joyner LA, Huggins J, Gilliland K, et al Fundamental dimensions of subjective state in performance settings: Task engagement, distress, and worry. Emotion. 2002;2(4):315–340. 10.1037/1528-3542.2.4.315 12899368

[34] Langner R, Steinborn MB, Chatterjee A, Sturm W, Willmes K. Mental fatigue and temporal preparation in simple reaction-time performance. Acta Psychologica. 2010;133(1):64–72. 10.1016/j.actpsy.2009.10.001 19878913

[35] Archer RP. Introducing the Minnesota multiphasic personality inventory-adolescent-restructured form (MMPI-ARF). European Scientific Journal, ESJ. 2016;12(10):147–153.

[36] Handel RW. An introduction to the Minnesota multiphasic personality inventory-adolescent-restructured form (MMPI-A-RF). Journal of Clinical Psychology in Medical Settings. 2016;23(4):361–373. 10.1007/s10880-016-9475-6 27752979

[37] Pavlenko V, Lutsyuk N, Borisova M. Correlation of the characteristics of evoked EEG potentials with individual peculiarities of attention in children. Neurophysiology. 2004;36(4):276–284. 10.1007/s11062-005-0019-1

[38] Niedermeyer E, da Silva FL. Electroencephalography: Basic Principles, Clinical Applications, and Related Fields, Nonlinear Dynamics. Lippincot Williams & Wilkins; 2014.

[39] Hramov AE, Maksimenko VA, Pchelintseva SV, Runnova AE, Grubov VV, Musatov VY, et al Classifying the perceptual interpretations of a bistable image using EEG and artificial neural networks. Frontiers in Neuroscience. 2017;11(674):1–18. 29255403 10.3389/fnins.2017.00674 PMC5722852

[40] Pavlov AN, Hramov AE, Koronovskii AA, Sitnikova YE, Makarov VA, Ovchinnikov AA. Wavelet analysis in neurodynamics. Physics-Uspekhi. 2012;55(9):845–875. 10.3367/UFNe.0182.201209a.0905

[41] Keirn ZA, Aunon JI. A new mode of communication between man and his surroundings. IEEE transactions on biomedical engineering. 1990;37(12):1209–1214. 10.1109/10.64464 2149711

[42] Lutsyuk N, Éismont E, Pavlenko V. Correlation of the characteristics of EEG potentials with the indices of attention in 12-to 13-year-old children. Neurophysiology. 2006;38(3):209–216. 10.1007/s11062-006-0048-4

[43] Rogers LJ, Zucca P, Vallortigara G. Advantages of having a lateralized brain. Proceedings of the Royal Society of London B: Biological Sciences. 2004;271(Suppl 6):S420–S422. 10.1098/rsbl.2004.0200 PMC1810119 15801592

[44] Kleinhans NM, Müller RA, Cohen DN, Courchesne E. Atypical functional lateralization of language in autism spectrum disorders. Brain Research. 2008;1221:115–125. 10.1016/j.brainres.2008.04.080 18555209 PMC2548303

[45] Herrington JD, Heller W, Mohanty A, Engels AS, Banich MT, Webb AG, et al Localization of asymmetric brain function in emotion and depression. Psychophysiology. 2010;47(3):442–454. 10.1111/j.1469-8986.2009.00958.x 20070577 PMC3086589

[46] Barry RJ, Clarke AR, Johnstone SJ. A review of electrophysiology in attention-deficit/hyperactivity disorder: I. Qualitative and quantitative electroencephalography. Clinical Neurophysiology. 2003;114(2):171–183. 12559224 10.1016/s1388-2457(02)00362-0

[47] Barry RJ, Johnstone SJ, Clarke AR. A review of electrophysiology in attention-deficit/hyperactivity disorder: II. Event-related potentials. Clinical Neurophysiology. 2003;114(2):184–198. 10.1016/S1388-2457(02)00363-2 12559225

[48] Luschekina E, Khaerdinova OY, Luschekin V, Strelets V. Interhemispheric differences in the spectral power and coherence of EEG rhythms in children with autism spectrum disorders. Human Physiology. 2017;43(3):265–273. 10.1134/S0362119717030112

[49] Santarnecchi E, Tatti E, Rossi S, Serino V, Rossi A. Intelligence-related differences in the asymmetry of spontaneous cerebral activity. Human Brain Mapping. 2015;36(9):3586–3602. 10.1002/hbm.22864 26059228 PMC6868935

[50] Milali MP, Sikulu-Lord MT, Kiware SS, Dowell FE, Povinelli RJ, Corliss GF. Do NIR spectra collected from laboratory-reared mosquitoes differ from those collected from wild mosquitoes? Plos One. 2018;13(5):e0198245 10.1371/journal.pone.0198245 29851994 PMC5978888

[51] Johnson SC. Hierarchical clustering schemes. Psychometrika. 1967;32(3):241–254. 10.1007/BF02289588 5234703

[52] Steinbach M, Karypis G, Kumar V. A comparison of document clustering techniques. In: KDD workshop on text mining. vol. 400. Boston; 2000. p. 525–526.

[53] Gomathi B, Suguna S. Comparison between clustering algorithms based on ontology based text mining techniques. International Journal of Advanced Research in Computer Science. 2014;5(7).

[54] Demŝar J, Curk T, Erjavec A, Gorup C, Hoĉevar T, Milutinoviĉ M, et al Orange: Data mining toolbox in Python. Journal of Machine Learning Research. 2013;14:2349–2353.

[55] Struyf A, Hubert M, Rousseeuw P, et al Clustering in an object-oriented environment. Journal of Statistical Software. 1997;1(4):1–30.

[56] Maksimenko VA, Lüttjohann A, Makarov VV, Goremyko MV, Koronovskii AA, Nedaivozov V, et al Macroscopic and microscopic spectral properties of brain networks during local and global synchronization. Phys Rev E. 2017;96:012316 10.1103/PhysRevE.96.012316 29347072

[57] Harmony T, Fernández T, Silva J, Bernal J, Díaz-Comas L, Reyes A, et al EEG delta activity: an indicator of attention to internal processing during performance of mental tasks. International Journal of Psychophysiology. 1996;24(1-2):161–171. 10.1016/S0167-8760(96)00053-0 8978441

[58] Harmony T. The functional significance of delta oscillations in cognitive processing. Frontiers in Integrative Neuroscience. 2013;7:83 10.3389/fnint.2013.00083 24367301 PMC3851789

[59] Klimesch W, Vogt F, Doppelmayr M. Interindividual differences in alpha and theta power reflect memory performance. Intelligence. 1999;27(4):347–362. 10.1016/S0160-2896(99)00027-6

[60] Raghavachari S, Kahana MJ, Rizzuto DS, Caplan JB, Kirschen MP, Bourgeois B, et al Gating of human theta oscillations by a working memory task. Journal of Neuroscience. 2001;21(9):3175–3183. 10.1523/JNEUROSCI.21-09-03175.2001 11312302 PMC6762557

[61] So WK, Wong SW, Mak JN, Chan RH. An evaluation of mental workload with frontal EEG. Plos One. 2017;12(4):e0174949 10.1371/journal.pone.0174949 28414729 PMC5393562

[62] Osaka M. Peak alpha frequency of EEG during a mental task: Task difficulty and hemispheric differences. Psychophysiology. 1984;21(1):101–105. 10.1111/j.1469-8986.1984.tb02325.x 6701238

[63] Başar E, Güntekin B. A short review of alpha activity in cognitive processes and in cognitive impairment. International Journal of Psychophysiology. 2012;86(1):25–38. 10.1016/j.ijpsycho.2012.07.001 22801250

[64] Zhang L, He W, He C, Wang P. Improving mental task classification by adding high frequency band information. Journal of Medical Systems. 2010;34(1):51–60. 10.1007/s10916-008-9215-z 20192055

[65] Palaniappan R. Utilizing gamma band to improve mental task based brain-computer interface design. IEEE Transactions on Neural Systems and Rehabilitation Engineering. 2006;14(3):299–303. 10.1109/TNSRE.2006.881539 17009489

[66] Vingiano W. Hemisphericity and personality. International Journal of Neuroscience. 1989;44(3-4):263–274. 10.3109/00207458908986206 2722416

[67] Pavlenko V, Chernyi S, Goubkina D. EEG correlates of anxiety and emotional stability in adult healthy subjects. Neurophysiology. 2009;41(5):337–345. 10.1007/s11062-010-9111-2

